# How type 1 fimbriae help *Escherichia coli* to evade extracellular antibiotics

**DOI:** 10.1038/srep18109

**Published:** 2016-01-05

**Authors:** Ima Avalos Vizcarra, Vahid Hosseini, Philip Kollmannsberger, Stefanie Meier, Stefan S. Weber, Markus Arnoldini, Martin Ackermann, Viola Vogel

**Affiliations:** 1 Laboratory of Applied Mechanobiology, Department of Health Sciences and Technology, ETH Zürich, 8093 Zürich, Switzerland; 2 Department of Biology, ETH Zürich, 8093 Zürich, Switzerland; 3 Department of Environmental Systems Science, ETH Zürich, 8092 Zürich, Switzerland; 4 Department of Environmental Microbiology, Eawag, 8600 Dübendorf, Switzerland

## Abstract

To survive antibiotics, bacteria use two different strategies: counteracting antibiotic effects by expression of resistance genes or evading their effects e.g. by persisting inside host cells. Since bacterial adhesins provide access to the shielded, intracellular niche and the adhesin type 1 fimbriae increases bacterial survival chances inside macrophages, we asked if fimbriae also influenced survival by antibiotic evasion. Combined gentamicin survival assays, flow cytometry, single cell microscopy and kinetic modeling of dose response curves showed that type 1 fimbriae increased the adhesion and internalization by macrophages. This was caused by strongly decreased off-rates and affected the number of intracellular bacteria but not the macrophage viability and morphology. Fimbriae thus promote antibiotic evasion which is particularly relevant in the context of chronic infections.

While internalization of microbes by immune cells often leads to their intracellular degradation, survival within host cells is a potent mechanism of bacterial virulence and antibiotic evasion^1^,^2^,^3^,^4^. The internalization of bacteria into eukaryotic host cells can be triggered either by the host cells, e.g. when immune cells recognize and actively phagocytose infecting bacteria, or by the bacteria, e.g. via release of proteins that enter host cells and promote bacterial internalization^5^,^6^. Although immune cells have optimized their ability to recognize and kill bacteria, some pathogenic bacteria specifically target cells of the immune system and achieve intracellular survival. The resulting failure of infected immune cells to clear bacteria, in combination with their highly migratory and tissue-invasive properties, can lead to a spread of the surviving bacteria and result in systemic and chronic infections^2^,^7^,^8^,^9^. Intracellular survival strategies of pathogens include escape from the phagosome into the cytosol and adaptation to their hostile surroundings by changing the biochemistry of their compartment as well as their own physiology^1^,^4^,^10^. Many pathogens have developed strategies to actively enter host cells and bacterial adhesion plays a critical role in optimizing survival chances^4^,^5^,^10^.

Bacterial adhesion to host cells precedes their internalization and is a critical step in the progression of bacterial infection caused by intracellular pathogens^5^,^7^,^11^. Among the large variety of bacterial adhesins that promote internalization into host cells, type 1 fimbriae are remarkably versatile virulence factors^3^,^12^,^13^,^14^,^15^,^16^,^17^,^18^,^19^,^20^. They mediate force activated catch bonds to mannosylated surfaces and cell receptors^12^,^14^, thus stabilizing the adhesion to host urinary epithelium under shear stress. Consequently, fimbriae were found to be essential for the virulence of many uropathogenic *E.coli* strains3,13,15,16^3^,^13^,^15^,^16^ and fimbriae also mediate adhesion to bacterial predators such as macrophages^18^. They were also found to correlate with increased survival inside their predators by affecting intracellular trafficking of *E.coli* in macrophagess^17^,^19^. In accordance with this, the expression of type 1 fimbriae is often associated with virulence of *E.coli* strains and loss of fimbriae often results in loss of virulence^2^,^6^,^20^,^38^. However, commensal *E.coli* also express fimbriae, although at lower levels^20^. Therefore, an understanding of the direct dependencies between bacterial adhesion and intracellular survival in phagocytes is of great medical relevance.

Phagocytes such as macrophages and neutrophils are immune cells specialized in the recognition and removal of foreign particles, which involves uptake and intracellular degradation by aggressive chemicals, and enzymes. Many bacteria have thus evolved strategies to prevent recognition and phagocytosis by immune cells^5^,^21^. It has been hypothesized that bacterial adhesins like fimbriae promote internalization into epithelial cells in which they may survive, but avoid adhesion to phagocytes, which are their predators^5^,^22^. However, some phagocytes have fimbriae-specific receptors that trigger fimbriae-specific internalization^17^,^18^. Surprisingly, fimbriae-dependent phagocytosis of bacteria was found to result in higher intracellular survival chances than other types of phagocytosis^17^,^23^. This suggests that fimbriae-mediated adhesion to macrophages helps *E.coli* to avoid clearance by the innate immune system^17^,^23^,^24^. Several studies compared survival of non-pathogenic and pathogenic bacteria, the latter of which often express additional virulence factors and may vary in the expression levels of these^15^,^20^. Different pathogens may therefore vary in their adhesion efficiency to macrophages and a quantification of the effects of adhesion efficiency on intracellular survival in macrophages is still lacking^22^. In particular, the question remains how the expression of fimbriae influences *E.coli* adhesion, survival, as well as the macrophage response to bacterial infection, i.e. if macrophages respond in a distinct way to fimbriated bacteria. Asking the question how fimbriation affects bacterial infection of macrophages and survival inside has additional clinical significance, since macrophages have been reported to release living pathogens which then continued to progress through their normal lifecycle^25^.

Our main goal was to quantify how adhesion by fimbriated *E.coli,* independent of other virulence factors, impacts intracellular survival in macrophages. Here, intracellular survival referred to bacteria that were internalized by macrophages and remained able to multiply after being extracted from the macrophage again. Internalization was assumed to occur via phagocytosis by the predatory immune cells and was expected to decrease the survival chances of *E.coli* since macrophages digest bacteria^26^. We first assessed bacterial survival chances for three different non-pathogenic strains of *E.coli*: a wild type strain (wt), a knock-out strain for type 1 fimbriae expression (∆fim), as well as a fimbriae overexpression strain (fim↑), which is isogenic with ∆fim but overexpresses fimbriae from the plasmid pSH2^27^,^28^,^29^. The pSH2 plasmid contains the whole fimbriae gene cluster and is a well-established expression system for investigations on overexpression of fimbriae^27^,^28^. We then asked if intracellular survival correlated with the efficiency of bacteria to adhere to macrophages and the number of bacteria per macrophage, i.e. the bacterial burden on a macrophage. To address this question, we established a dose-response curve for bacterial adhesion to macrophages. We inhibited the functionality of fimbriae and the bacterial burden with two approaches. We used fimbriae-specific inhibitors to inhibit adhesion and cytoskeletal inhibitors to inhibit phagocytosis. The bacteria to macrophage ratios were systematically varied in a range from 0.5 to 32. These ratios have often been referred to as multiplicities of infection (MOI)^17^,^19^,^30^ which implicates, sometimes incorrectly, a linear relationship between bacterial concentration and host cell infection. However, such linear relationship might not exist. We furthermore asked if we could identify a mechanistic model underlying bacterial adhesion to macrophages that could quantify the adhesion parameters in dependence of fimbriae expression. Finally, we asked if expression of fimbriae and bacterial burden influenced the macrophage morphology in response to bacteria. Such morphological changes could give indications to a fimbriae-specific activation or suppression of a characteristic macrophage response. The macrophage morphology was thus quantified regarding macrophage spreading, viability, proliferation and surface adhesion.

## Results

### Overexpression of type 1 fimbriae yielded more *E.coli* survivors inside of macrophages

**Figure 1:**
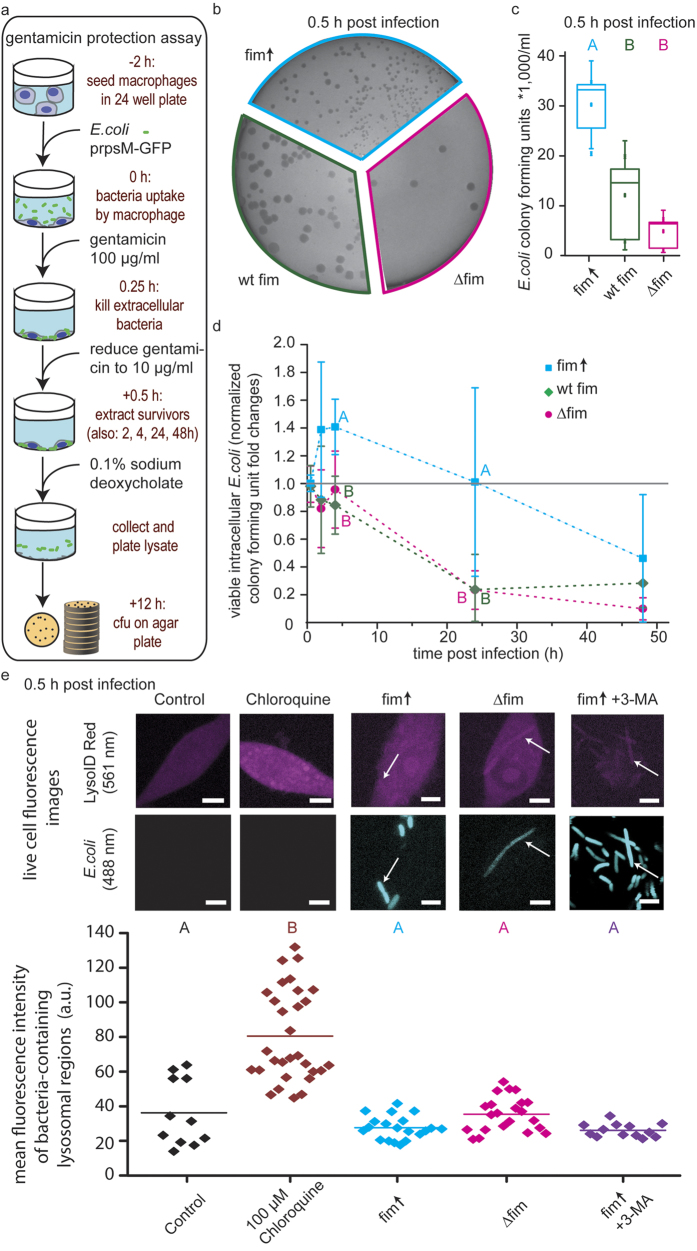
*E.coli* overexpressing type 1 fimbriae yielded more intracellular survivors in macrophages. (**a**) Timeline of the gentamicin assay for the measurement of intracellular survivors. Gentamicin was applied as a 15 minute pulse of 100 μg/ml to kill extracellular bacteria and was then reduced to 10 μg/ml for long term co-cultivation. (**b**) Fimbriae expression affected the number of viable bacteria extracted from macrophages. A composite image of 3 randomly chosen agar plates is shown from macrophage lysates after incubation with fim↑, wt fim and ∆fim, respectively, using the protocol as sketched in (a). (**c**) Total colony forming units recovered from gentamicin protection assays 0.5 hours post infection showed increased survivor numbers of fim↑ compared to wt fim and ∆fim bacteria, using the protocol as sketched in (a). RAW macrophages were seeded to 10^5^ cells/cm^2^ and incubated with the three different *E.coli* strains, respectively, at a bacteria-to-macrophage ratio of 10:1. Box plot whiskers indicate the S.D. The variance of population means was analyzed using a one way ANOVA. Upper-case letters mark significant differences based on post hoc Tukey and Bonferroni tests. Pairs of samples that have no letter in common have significantly different means at p < 0.01; i.e., samples with the label A are significantly different from samples with the label B. (**d**) Fold changes of colony forming units, normalized to 0.5 hours post infection, respectively, indicated higher survival chances of fim↑ bacteria compared to wt fim and ∆fim. Error bars are S.D. The variance of population means was analyzed in the same way as in (c). (**e**) Staining for lysosomal acidification did not yield significant differences in the LysoID Red fluorescence between fim↑ and ∆fim (p = 1). Scale bar is 5 μm. fim↑, fimbriae overexpression strain; wt, fimbriae wild type strain; ∆fim, fimbriae knockout strain; 3-MA, 3-methyladenine; h, hours

Our first goal was to investigate the specific impact of fimbriation on the survival chances of *E.coli* inside macrophages - independently of other virulence factors. The intracellular survival was quantified in gentamicin protection assays for up to 48 hours post infection (Fig. 1a, 1d). The exposure to the antibiotic gentamicin ensured that all bacteria that had not been internalized by the phagocytes were rendered incapable of replication. Bacterial survivors were quantified from single colonies based on their ability to replicate on agar plates after macrophage lysis (Fig. 1a,b). For a fimbriae-mediated survival advantage, we expected to find different behavior of bacterial clearance by macrophages, i.e. differences in the temporal dynamics of colony forming units. This would not require differences in the initial survivor numbers. However, we found large differences in the absolute numbers of surviving bacteria from the first time point onwards. The total numbers of colony forming units extracted from macrophages were 6-fold increased for fim↑ as opposed to ∆fim and 3-fold increased to wt, while using the same bacteria-to-macrophage ratio of 10:1 for all three strains (Fig. 1c).

Normalization to the absolute numbers of colony forming units to the 0.5 hour time point showed that *E.coli* overexpressing fimbriae were cleared less efficiently from macrophages (Fig. 1d). The finding on normalized survival is in good agreement with previous studies^17^,^23^, while the absolute numbers indicated that more bacteria were internalized from the beginning on when they overexpressed fimbriae. This had to the best of our knowledge not been reported before. Furthermore, a pronounced increase in intracellular viable fim↑ bacteria was observed 2 and 4 hours post infection, indicating an inefficient clearance of these bacteria, and their replication inside macrophages (Fig. 1d). After 4 hours post infection, a decrease in intracellular survivors was observed also for fim↑, although the fraction of intracellular fim↑ survivors was still significantly higher than for wt and ∆fim (p = 2.45*10^−4^ and 2.45*10^−3^, respectively) as determined by a one-way ANOVA at p  <  0.01. Furthermore, fim↑ survived within macrophages to higher levels for more than 48 hours (Fig. 1d). While this assay showed that more bacteria survived when they overexpressed fimbriae, it was not possible to differentiate whether the presence of fimbriae yielded overall higher numbers of infected macrophages with low bacterial burden or just increased the bacterial burden and fraction of survivors in a small macrophage subpopulation (Fig. 1d). To test if differences in lysosomal acidification could explain the observed differences in survivors, we performed a series of lysosomal assessments using the fluorescent dye LysoID^31^ (Fig. 1e). While Chloroquine, a known activator of the lysosomal pathway^31^ showed a clear increase in LysoID signal intensity, this was not observed after incubating macrophages with either fim↑ or ∆fim (Fig. 1e). Since a parallel pathway for bacterial intracellular trafficking by autophagy was reported^19^, we blocked autophagy with the inhibitor 3-methyladenine (3-MA) to test if this would increase the flux through the lysosomal pathway. However, the LysoID Red fluorescence intensity remained unaffected by treatment with 3-MA (Fig. 1e) when measuring the bacteria-containing regions. This indicated that blocking autophagy at 0.5 hours post infection did not interfere with the lysosomal acidification. Based on these data, we concluded that differences in lysosomal acidification did not contribute to higher survival chances of fim↑.

### Adhesion to macrophages and bacterial burden were strongly increased for type 1 fimbriae overexpressing *E.coli*

**Figure 2:**
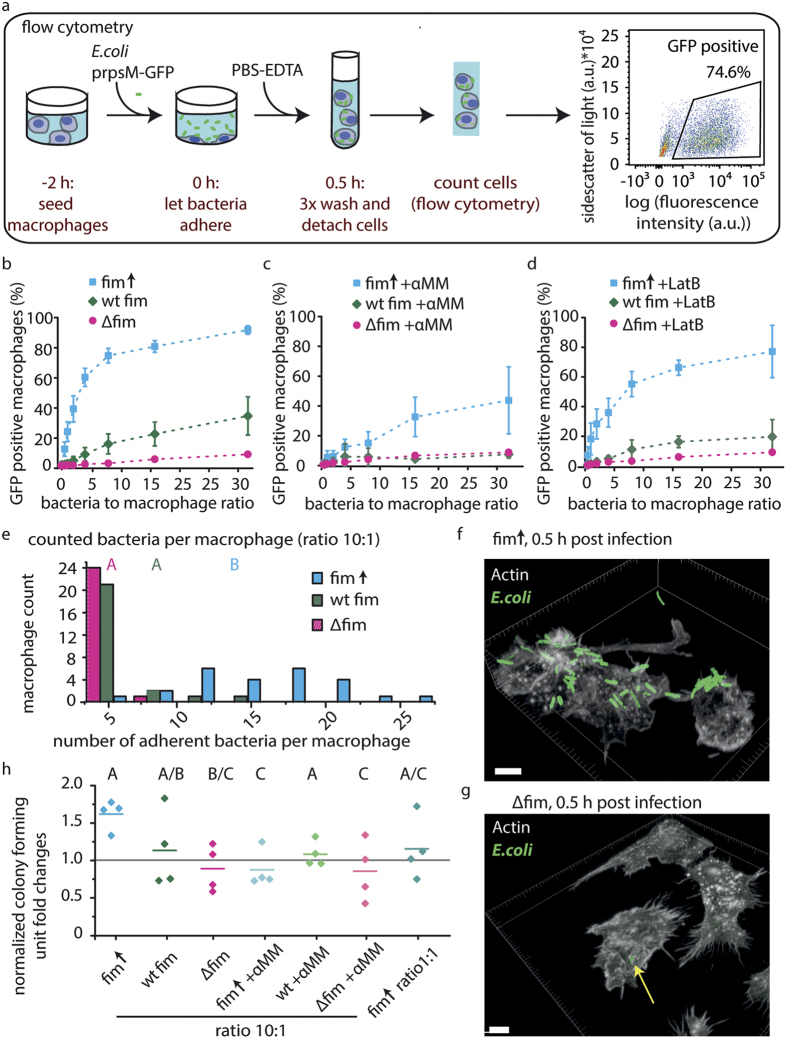
Fimbriae overexpression yielded higher adhesion efficiency, bacterial burden and intracellular survival of *E.coli* in macrophages. (**a**) Timeline of the flow cytometric determination of the bacterial adhesion efficiency to macrophages. (**b**) Expression of type 1 fimbriae changed the adhesion efficiency of *E.coli* to macrophages. Macrophages were incubated with *E.coli* fim↑, wt fim and ∆fim, respectively, at bacteria-to-macrophage ratios from 0.5 to 32. Error bars are S.D. from n = 8 independent experiments. (**c**) Higher adhesion efficiency depended on functional type 1 fimbriae, as the mannose analogue αMM decreased adhesion of fim↑ but not ∆fim. (**d**) Treatment of macrophages with LatB yielded less GFP-positive macrophages. (**e**) The bacterial burden on macrophages was higher for fim↑ compared to ∆fim. Images of 25 randomly chosen macrophages were analyzed for total counts of adherent bacteria. (**f**) Macrophages 0.5 hours post infection with fim↑ and (**g**) ∆fim at a ratio of 10:1 bacteria per macrophage, respectively. GFP-expressing bacteria are colored in green, actin-binding phalloidin is colored in grey. One adherent ∆fim bacterium is indicated by the yellow arrow. Scale bar is 5 μm. (**h**) αMM decreased survivors of fim↑ inside macrophages. Variance of population means was analyzed using a one way ANOVA. Upper-case letters mark significant differences based on a post hoc Tukey and Bonferroni test. Pairs of samples that have no letter in common have significantly different means at p < 0.05; i.e., samples with the label A are significantly different from samples with the label B/C, but not from samples with the label A/B. cfu, colony forming units; fim↑, fimbriae overexpression strain; wt, fimbriae wild type strain; ∆fim, fimbriae knockout strain; GFP, green fluorescent protein; αMM, alpha-methyl mannosepyrannoside; LatB, LatrunculinB; h, hours.

We next quantified infection efficiency and bacterial burden, i.e. the bacteria per macrophage on the single cell level. Towards this end, we investigated how fimbriae affected bacterial adhesion to macrophages using GFP-expressing *E.coli* of the fim↑, wt and ∆fim strains. Macrophages that bind or internalize GFP-expressing bacteria were detected by their fluorescence signal using flow cytometry (Fig. 2a). To quantify the bacterial burden on macrophages, the ratio of GFP-expressing bacteria to macrophages was systematically varied from 0.5 to 32, and the relative number of GFP-positive macrophages, i.e. the population of macrophages associated with bacteria, was quantified by flow cytometry (Fig. 2a). Serum-free media was used for 0.5 hours during adhesion, whereas all other cultivation was performed in media supplemented with serum to prevent nutrient limitation of macrophages. A potential effect of serum on adhesion efficiency was tested by using serum-containing media also during the adhesion assay (Supplementary Fig. S1a) and yielded the same trend of adhesion behavior.

The percentage of GFP-positive macrophages incubated with fim↑ was strongly increased compared to ∆fim after the same incubation time of 0.5 hours. Notice that we carefully ensured that this comparison was made for the same bacteria to macrophage ratio of 10:1 (Fig. 2b). Consistent with this, we found a larger non-fluorescent macrophage population for ∆fim, indicating that adhesion between ∆fim and macrophages is less efficient (Supplementary Fig. S1d). The wt strain showed an intermediate adhesion efficiency between fim↑ and ∆fim (Fig. 2b), consistent with stochastically distributed levels of fimbriae expression in bacterial wild type populations^32^.

Since the efficiency of macrophage infection showed a clear dependency on fimbriae expression and adhesion to macrophages (Fig. 2b), we next inhibited the adhesion efficiency of bacteria to macrophages. The molecular basis of fimbriae mediated adhesion is their binding to mannose sugars on host cell receptors by the mannose-specific lectin FimH. To block fimbriae-specific adhesion, a competitive inhibitor for the FimH lectin, the mannose analogue alpha-methyl-pyrannoside (αMM), was added to the medium^12^,^18^,^28^. Indeed, αMM strongly reduced adhesion of the fim↑ strain as well as of the wt strain, indicating that higher adhesion of the wt and fim↑ strains was due to the presence of functional fimbriae (Fig. 2c). Consequently, αMM had no effect on adhesion of ∆fim bacteria, as expected from its known specificity for blocking fimbriae-mediated macrophage adhesion (Fig. 2c). We also used Latrunculin B (LatB), a potent inhibitor of actin polymerization to inhibit actin-dependent internalization of bacteria. Incubation with LatB prevented internalization of bacteria while not inhibiting the adhesion to macrophages per se. LatB treated macrophages were unable to retain bacteria with the help of actin driven protrusions, since LatB blocks the actin polymerization required for this process, and a smaller GFP-positive macrophage population was observed in all LatB treated samples (Fig. 2d). These results showed that fimbriae increased the efficiency by which bacteria bind to and are taken up by macrophages, thereby also suggesting a larger bacterial burden. It should be noted that all data from the flow cytometry assay, excluding those with LatB treatment, will inherently show a mix of surface-attached and internalized bacteria. We did not see any differences in fluorescence intensity between surface-attached and internalized bacteria (Supplementary Fig. S1b), which is in agreement with the constitutive expression of GFP from the *E.coli rpsM* promoter.

To test if bacterial burden itself could contribute to intracellular survival, we quantified the bacterial burden by counting bacteria on macrophages using confocal fluorescence microscopy 0.5 hours post infection at a bacteria-to-macrophage ratio of 10:1 (Fig. 2e, Supplementary movies M1 and M2). For a bacteria-to-macrophage ratio of 10:1, more fim↑ bacteria adhered per macrophage than for ∆fim (Fig. 2e–g, Supplementary movies M1 and M2).

Consistently, the intensity distribution of GFP-positive macrophage populations from the flow cytometric data showed a shift towards higher intensities for fim↑ compared to ∆fim. This intensity shift indicated several stably adherent bacteria per macrophage (Supplementary Fig. S1c) and was also confirmed by the number of bacteria per macrophage (Fig. 2e). We thus showed that fimbriation resulted in an increased bacterial burden per macrophage. Comparison between experiments with the differently fimbriated bacteria showed the same, i.e. that expression of fimbriae led to a larger infected macrophage fraction, and increased bacterial burden per macrophage.

To clarify whether intracellular survival and adhesion efficiencies were functionally linked, we next asked if inhibiting bacterial adhesion via fimbriae would have consequences on intracellular survival. The gentamicin protection assay was repeated under the influence of inhibitors (Fig. 2h, Supplementary Fig. S1d) to test if the inhibitors could modulate intracellular survival chances in a similar manner since they changed adhesion behavior. Incubation with LatB however, did not yield any colony forming units (Supplementary Fig. S1d). This finding is consistent with the implicit assumption of the assay that only intracellular bacteria can survive antibiotic treatment. Bacterial adhesion to the outer plasma membrane of macrophages alone is thus not sufficient to protect bacteria from the effect of extracellular antibacterial drugs. Interfering with the fimbriae-mediated adhesion of bacteria to macrophages via the monomannose analogue αMM reduced intracellular colony forming units of fim↑ and wt fim, but not of ∆fim (Fig. 2h). This is consistent with the competitive binding of αMM for the FimH binding pocket^12^,^18^. Taken together, incubation with αMM reduced intracellular colony forming units of fim↑ bacteria to those levels of ∆fim. The fimbriae inhibitor αMM thus blocked the enhanced infection of macrophages by fimbriated bacteria (Fig. 2c). To test whether this effect was due to increased bacterial burden for infections with fim↑, we tested bacterial survival with a bacteria-to-macrophage ratio of 1:1 instead of 10:1 (Fig. 2h). The ratio of 1:1 for fim↑ was motivated by comparing the GFP-positive macrophage populations of the fim↑ at a ratio of 1 with that of the ∆fim strain at a ratio of 10:1. The tenfold increase of ∆fim bacteria numbers yielded similarly large GFP-positive macrophage population than a ratio of 1 fim↑ bacterium per macrophage. We found that despite an apparent reduction of the intracellular survivors, the levels of intracellular survivors of fim↑ at a bacteria-to-macrophage of 1:1 were not significantly different from the fim↑ and ∆fim strains at a ratio of 10:1 (p = 1, and p = 1, respectively), as determined by a one-way ANOVA at p < 0.05 (Fig. 2h). The results derived from quantifying the modulation of bacterial burden therefore suggest that functional fimbriae have a larger impact on intracellular survival than the sole effect of bacterial burden, and finding that had not been shown before.

### Quantification of infection doses and of the influence of inhibitors on adhesion efficiencies

**Figure 3:**
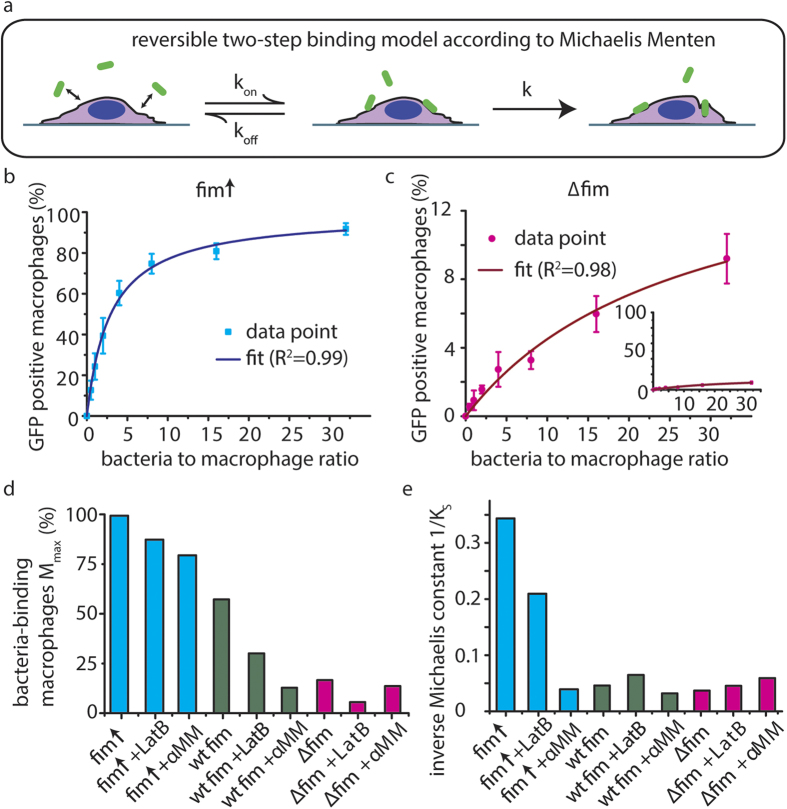
A quantitative model adapting Michaelis Menten kinetics to bacteria-macrophage interactions allowed to estimate infection doses and rate constants. (**a**) Formalized adhesion sequence between macrophages and bacteria according to Michaelis Menten kinetics. (**b**) Experimentally derived data (error bars are S.D.) and Michaelis Menten fit (given as solid line) are shown for fim↑. (**c**) Experimentally derived data and Michaelis Menten fit (given as solid line) are shown for ∆fim. Inset graph shows the full range to enable a comparison with the graph in b. (**d**) Michaelis Menten model predictions of the M_max_ parameter showed the maximal relative number of macrophages that can bind to *E.coli* fim↑, wt, and ∆fim. (**e**) Fitting to the Michaelis Menten model yielded the inverse of the adhesion constant (1/K_s_), and thus the kinetic rate constants for the adhesion of *E.coli* fim↑, wt, and ∆fim strains to macrophages. Error bars are S.D.; k_on_, reversible adhesion on-rate; k_off_, reversible adhesion off-rate, k, irreversible adhesion rate; 1/K_s_, inverse Michaelis Menten constant; M_max_, maximum fraction of bacteria-bound macrophages; fim↑, fimbriae overexpression strain; wt, fimbriae wild type strain; ∆fim, fimbriae knockout strain; GFP, green fluorescent protein; αMM, alpha-methyl mannosepyrannoside; LatB, Latrunculin B.

To further increase our mechanistic understanding of the *E.coli* binding process to macrophages, we evaluated models that could quantify the key features of bacterial adhesion. The data points from the flow cytometric determination of adhesion efficiencies (Supplementary Fig. S1b, S1c) were fit to two mathematical models. The best fit was obtained using a model inspired by Michaelis Menten kinetics (Fig. 3a–c, eq. (1)) that describes enzyme-substrate reactions. The model describes an adhesion process with a first step under an equilibrium assumption of adhesion and unbinding and a second irreversible and thus rate-limiting step (Fig. 3a, Supplementary Information, eq. (1)–(8)). By adapting the Michaelis Menten kinetics to bacteria-macrophage adhesion (Fig. 3a, Supplementary Information, equations (2)–(8)), bacterial adhesion could be described by two sequential steps: first, a reversible step of initial adhesion with an on-rate and an off-rate that leads to a bacteria-macrophage equilibrium complex. In principle, this complex is transient and can dissociate again. The second step is an irreversible and thus rate limiting step leading to stable adhesion of bacteria and their internalization. The overall uptake rate R(x) of bacteria by macrophages is described as function of the percentage of macrophage population that can bind bacteria M_max_ (Fig. 3d), the ratio of bacteria to macrophages x, and the Michaelis Menten constant K_s_ (Fig. 3e) which is given by the ratio of the off-rate and on-rate for the first reversible adhesion step:


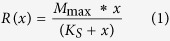


For a given on-rate, an increase in 1/K_s_ is a quantitative measure for the affinity of bacteria to macrophages as it corresponds to a decreased off-rate for the initial adhesion equilibrium (Fig. 3a). The value of M_max_ is proportional to the rate constant of the second, rate-limiting step leading to internalization and thus determines the infection efficiency by the maximum possible number of infected macrophages. The parameters M_max_ and the adhesion constant obtained from the fits followed a similar trend as the experimentally determined adhesion efficiencies for the fim↑ and ∆fim strains (Fig. 3b–d). A detailed description of two alternate models can be found in the Supplementary information, including tests for the quality of a second model for an alternative adhesion mechanism assuming one irreversible adhesion step (see Supplementary equations (9)–(12), Supplementary Fig. S2a, S2b).

In summary, the Michaelis Menten-derived two-step model approximates the uptake kinetics and thus provides insights how the same numbers of bacteria that surround macrophages yield different infection efficiencies (Fig. 3b–d). According to this kinetic model, type 1 fimbriae lead to a strongly decreased dissociation rate due to a decreased off-rate of initial binding. This shifts the equilibrium towards the bound state (Fig. 3b,e), which in turn increases the likelihood of internalization via the second, rate-limiting step. Thus, the expression of fimbriae shifted the initial equilibrium of adhesion strongly to the side of stable adhesion. While the experiments here were conducted without flow, the presence of flow is expected to additionally decrease the off-rate since the FimH-mannose complex can form force-activated catch bonds^12^.

### Exposure to bacteria substantially changed the macrophage morphology: this phenotypic response was not further upregulated by fimbriation or bacterial burden

**Figure 4:**
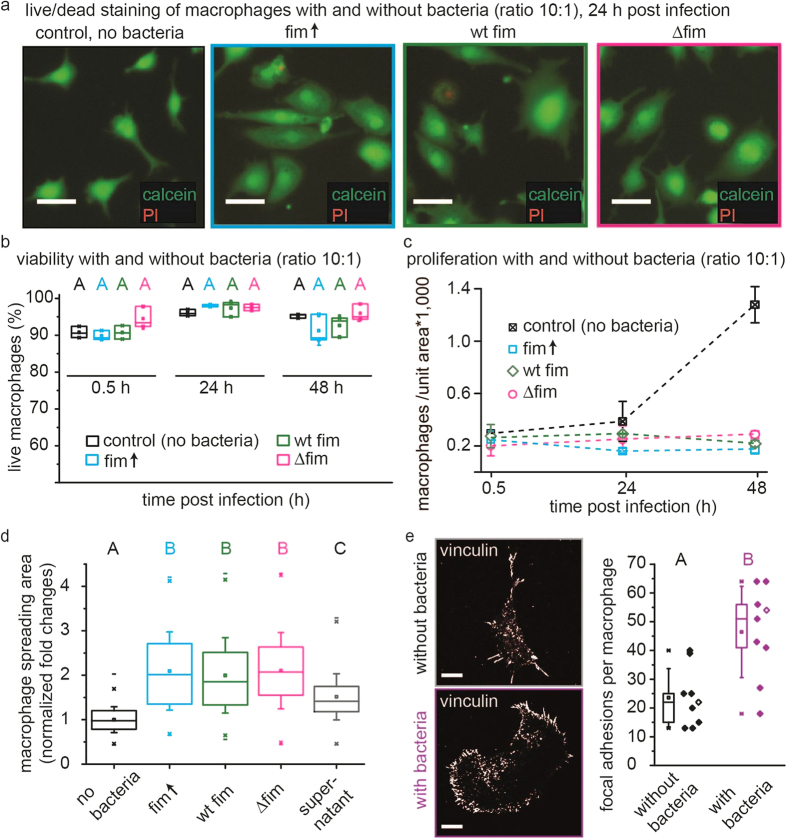
Substantial changes in the macrophage morphology occur upon exposure to *E.coli* and this response is neither affected by bacterial fimbriae expression nor the bacterial burden. (**a**) Macrophages adhering to fibronectin coated glass surfaces, 24 hours post incubation without bacteria, with *E.coli* fim↑, fim wt and ∆fim at a bacterial burden of 10:1, respectively. Scale bar is 20 μm. (**b**) Viability of macrophages was independent of fimbriae expression and was assessed by membrane permeability assays using calcein (live cells) and propidium iodide (dead cells). (**c**) Macrophage proliferation was irreversibly inhibited post infection. (**d**) Cell surface areas were assessed by thresholding fluorescent micrographs of macrophages stained with the calcium-chelating live dye calcein 24 hours post infection. Cells in the control were not exposed to *E.coli*; supernatant refers to bacteria-conditioned media. Error bars are S.D. from n = 3 independent wells with 10^5^ cells/well respectively in (**b–c**); Variance of population means was analyzed using a one way ANOVA. Upper-case letters mark significant differences based on a post hoc Tukey test. Pairs of samples that have no letter in common have significantly different means at p < 0.01; i.e., samples with the label A are significantly different from samples with the label B. (**e**) Macrophage surface interaction was assessed by immunostaining vinculin and counting of focal adhesion complexes at the cell periphery, and showed increased numbers of focal adhesion complexes post infection. Two randomly chosen images from vinculin immunostainings are shown on the left. Scale bar is 10 μm. The box plot shows focal adhesions per cell which were identified manually by their elongated spike shape from binarized images and counting, n = 9. Data points belonging to the pictures on the left have no fill. Variance of population means was analyzed using a one way ANOVA and post-hoc Tukey and Bonferroni test. Groups for statistical significance are given by upper case letters; different letters indicate that population means are significantly different at p < 0.001. fim↑, fimbriae overexpression strain; wt, fimbriae wild type strain; ∆fim, fimbriae knockout strain; h, hours.

Finally, we asked whether the increased bacterial survival was a consequence of differences in macrophage viability. The viability of macrophages was assessed by staining with the calcein dye, which stains only cells with intact membranes (Fig. 4a). Only the dead cells stain with propidium iodide, since it can enter only those cells with lysed or permeable membranes. This assay yielded two important insights: First, the viability of macrophages incubated with fim↑ for 24 hours was not significantly different (p = 1) from the viability of macrophages incubated with ∆fim for 24 hours as determined by a one-way ANOVA at p < 0.001 (Fig. 4b). Second, we also observed that the number of macrophages per field of view was larger for cells in non-infected control, while the cell spreading area was smaller. The macrophage proliferation during 48 hours of incubation with and without bacteria was investigated by counting live cells over time. This analysis showed that proliferation was indeed inhibited when macrophages were incubated with bacteria (Fig. 4c). The results suggest that macrophages increased their interaction with the surface by increased spreading after encountering bacteria.

To investigate if the adhesion strength to the surface was also increased and if fimbriae played a role in this process, we investigated the morphology of surface-adherent macrophages by immunostaining the protein vinculin. Changes in the morphology and spreading of macrophages have been described previously^33^,^34^,^35^ and are instrumental for their role in anti-inflammatory behavior as well as infection-associated inflammation. Furthermore, it was recently found that increasing the formation of long-lived focal adhesions had an impact on intracellular trafficking and survival of pathogens^35^. We tested these long-lived focal adhesions and whether the tensile state by which macrophages are anchored to the substrate is altered upon exposure to bacteria, by immunostaining for vinculin (Fig. 4d). Vinculin is recruited to stretched talin within focal adhesions and has also been found in immune cell podosomes, which mediate migration and invasion inside tissues as immune cells screen for foreign antigens^8^,^36^. Once exposed to bacteria, macrophages increased in size (Fig. 4a), whereby the spreading area increased by a factor of 2 after 24 hours (Fig. 4e). This occurred independently of the state of bacterial fimbriation. Infected macrophages also contained more vinculin-positive focal adhesion complexes per macrophage, indicative of stronger surface interaction (Fig.4d). The increase in focal adhesion complexes was again independent of whether the bacteria were fimbriated or not (Fig. 4d). Taken together, macrophage infection significantly increased the number of focal adhesions (p = 2.2*10^−5^), surface spreading area, and macrophage viability, but these effects were consistently independent of the bacterial burden.

### Discussion and Physiological Significance

While bacterial adhesion via type 1 fimbriae helps bacteria to invade host cells^3^,^6^,^15^,^37^, the same adhesins also pose a risk for bacteria when contributing to their recognition and clearance by phagocytic cells^18^,^24^. Remarkably, fimbriation not only promotes the adhesion to phagocytes, but also the bacterial survival in macrophages^2^,^17^,^19^,^23^. However, fimbriae-mediated intracellular survival has been discussed controversially in the literature. Baorto *et al.* (1997) and Amer *et al.* (2005) found fimbriae-dependent effects on survival, while e.g. Hamrick *et al.* (2000) concluded that fimbriae did not impact intracellular survival in macrophages. However, in the latter study, 12 experiments with a positively sloped killing curve were excluded from the analysis, indicating a heterogeneity in the macrophage population. A potential heterogeneity is also confirmed by the authors finding that the least bactericidal macrophages bound more bacteria per macrophage which is in good agreement with our findings here. In a later study by the same authors fimbriae-specific survival was furthermore reported for antibiotics-independent protection assays. To gain insights into the principles underlying survival-benefits of fimbriated bacteria, we used non-pathogenic *E.coli* strains that differed only in their fimbriae expression levels. By quantifying the number of bacteria per macrophage compared to non-fimbriated bacteria at the same dose (Fig. 2e), we could show here that fimbriae strongly increase adhesion of *E.coli* to macrophages (Fig. 2b) by decreasing the probability of bacterial unbinding upon contacting the macrophage surface (Fig. 3b. As a consequence, *E.coli* accumulates inside macrophages faster to high bacterial doses (Fig. 1c,d), and increases its uptake probability even at low doses (Fig. 2b). Our data now clarify the perceived controversy, as we can show that the above mentioned inconsistencies are a consequence of increased adhesion and internalization of fim↑ compared to ∆fim.

From the quantification of the bacterial burden on macrophages, we conclude that the fimbriae-mediated burden alone did not significantly increase the chance of a phagocytosed bacterium to survive significantly (p = 1), as determined by a one-way ANOVA at p < 0.05 (Fig. 2h). Instead, fimbriation increased the uptake of *E. coli* into the intracellular niche such that more bacteria survive within a macrophage when compared to the same exposure to non-fimbriated bacteria. In addition to the increased survival, we also observed an increase of fim↑ numbers inside macrophages after their uptake. This contributed to enhance the survival chances of fim↑ (Fig. 1d). Seeing that a short incubation time of 0.5 hours was sufficient to result in large survival differences between fimbriated and non-fimbriated bacteria, we suggest that accelerating stable adhesion and internalization ensures faster removal of bacteria from the environment. Taken together we can furthermore conclude that fimbriation is a favorable asset for *E.coli* to survive in the presence of extracellular antibacterial drugs and that this occurs by upregulating the FimH-mediated binding efficiency to macrophages.

Type 1 fimbriae are required virulence factors in urinary tract infections and intracellular biofilm formation^3^,^11^,^15^,^16^, suggesting various physiological significant implications. First, we identified the kinetic mechanism behind the fimbriae-mediated increase of the internalization rate and consequently of the bacterial burden on host cells. This illustrates that the enhanced bacterial adhesion to host cells constitutes a survival benefit. Moreover, higher expression levels of fimbriae are found in pathogenic strains^15^,^20^,^38^, even though commensal *E.coli* strains also express fimbriae^32^. However, the switching rates to the on-state of expression were found to be higher in pathogenic than in commensal strains^20^. Consistently, we found that fimbriae-overexpressing bacteria increased infection efficiency of macrophages even at very low infection doses of bacteria (Fig. 2b), highlighting how the upregulation of fimbriae-expression might tune virulence.

Second, we show here that an adapted Michaelis Menten kinetic model could fit the bacteria-macrophage interactions (Fig. 3b,c, Supplementary Fig. S2). This strongly suggests that bacterial binding to macrophages is not an irreversible one-step process. The finding that M_max_ is less than 100% can be explained with the necessary transition between the first reversible adhesion step and an irreversible second internalization step. According to the Michaelis Menten kinetics, M_max_ thus depends on the rate constant k of the internalization step exclusively. For a very high off-rate of the first binding step, the probability of reaching the equilibrium will be so low that the infection of macrophages does not reach saturation. Another possible explanation would be that these macrophages do not have mechanical contact to bacteria at all, which is however unlikely, given that the concentration of bacteria was the same for all strains. The kinetic model described here suggests ligand-dependent off-rates as alternative explanation. Such quantification of dissociation constants is particularly useful to quantify host cell recognition and host receptor adhesion efficiencies.

Third, and perhaps most importantly in the context of bacterial survival, internalized bacteria are shielded from treatments with antibacterial drugs that cannot pass the plasma membrane of macrophages. This shielding effect against such drugs that do not penetrate host cell membranes was noticed already in the 1960s^39^ and is broadly exploited since then to distinguish surface-bound from internalized bacteria^40^. The use of antibiotics that accumulate also inside host cells can reduce this shielding effect, but is influenced by e.g. the intracellular pH and the location of the bacteria in compartments^41^,^42^. Additionally, the intracellular accumulation of antibiotics was reported to affect the phagocyte phenotype in some cases^43^,^44^. Here, the extracellular antibiotic gentamicin was used to distinguish intra- and extracellular bacteria. The correlation of bacterial adhesion kinetics and survival has also significant implications for therapeutic drug applications: a kinetic advantage for entry into the intracellular niche is an intriguing survival benefit for antibiotics-sensitive bacteria such as we have used here^45^,^46^,^47^. The observed survival in macrophages without inducing macrophage apoptosis^2^,^6^ (Fig. 1d and 4b) can be a powerful strategy of bacterial pathogens. This is of particular relevance when migratory and tissue-invasive host cells such as macrophages can be used as transport vehicles. Using host cells as vehicles, bacteria can overcome epithelial barriers and invade deep tissues and spread throughout the host. Spreading of bacteria can also lead to the establishment of persistent and chronic infections^2^,^6^,^9^,^11^ and is especially relevant when an infection with bacteria does not lead directly to host cell death - as observed in this study as well as for many clinical isolates^2^,^6^.

In summary, our data show that the virulence portfolio of fimbriated bacteria has to be extended, namely that fimbriation also affect the rate of internalization into their own predators, namely macrophages. The duration as well as frequency of antibiotic exposure have a substantial impact on the survival and persistence of virulent bacteria in antibiotics-containing environments^48^,^49^. Since killing of extracellular and unrestrictedly growing bacteria by antibiotics and antibacterial drugs occurs fast, usually in the course of one to few hours^50^,^51^, escaping from an antibiotics-containing environment is crucial for bacteria that are under the selective pressure of antibacterial drugs^10^,^46^,^49^. *E. coli* thereby profits from optimized adhesion, already in the absence of more costly and time consuming expression of e.g. resistance genes. This is particularly relevant for the failure of antibacterial drugs leading to untreatable persistent and chronic infections. The increased infection efficiency, which caused the observed differences in bacterial burden, can be a powerful tool for pathogenic strains with additional survival-beneficial virulence factors. Fimbriae can thus serve as an infection accelerator which then allows shielding from extracellularly applied drugs. From a bioengineering or immune therapy perspective, fimbriation of drug or gene-delivery systems could be exploited to deliver cargo more efficiently to immune cells without inducing cell death.

## Methods

### Macrophages

We used the immortalized monocyte-derived murine cell line RAW264.7 (ATCC TIB-71, Middelesex, USA) which has been widely employed in the study of host-pathogen interactions as it provides a stable genetic background and constant expression of macrophage surface markers. RAW264.7 macrophage-like cells were cultivated in RPMI (Gibco 1640, Zug, Switzerland) supplemented with 10% FBS (Biowest S1810, Nuaille, France), 2 mM Glutamine (Gibco 25030), and 25 mM HEPES (Biowest L0180).

### Bacteria

Bacterial strains employed were the *E.coli* K12 derivate MG1655, which is the parental strain of AAEC191. The AAEC191 strain is chromosomally knocked out for type 1 fimbriae^28^,^29^ and the same strain was also reconstituted for fimbriae overexpression with the constitutively expressed plasmid pSH2 encoding the complete fim operon. MG1655 was employed here as *E.coli* wt. Constitutive GFP expression was achieved by transforming all strains with the P_*rpsM*_-GFP vector from a genomic fusion library^52^. *E.coli* strains were grown in LB media and diluted in serum-free cell culture medium prior to experiments after overnight growth to an optical density of 0.1.

### Bacterial survival and macrophage viability assays

All experiments were based on a 0.5 hour incubation of surface adherent macrophages with bacteria at 37 °C in which phagocytosis was allowed to occur unperturbed. Intracellular bacterial survival was assessed by standard gentamicin protection assays^17^,^19^,^23^. Briefly, 1 × 10^5^ RAW264.7 cells per well were seeded in tissue culture treated 24-well-plates (TPP 92424) in serum-free media (Sigma 5921) supplemented with 25 mM HEPES, and 2 mM L-Glutamine. Bacteria transformed with the P_*rpsM*_-GFP promoter fusion construct^53^ were added in the respective bacteria to macrophage ratio. Synchronization of infection occurred by centrifugation for 1 minute at 395 g and subsequent incubation at 37 °C for 0.5 hours. Media was then replaced for 0.25 hours to serum free media supplemented with 100 μg/ml gentamicin (Sigma 48760-1G-F) and after that to media supplemented additionally with 10% FBS and 10 μg/ml gentamicin. Cultivation was continued until each indicated time point when cells were lysed with 0.1% sodium deoxycholate and cell lysates plated on agar plates for quantification of colony forming units (cfu). Normalized intracellular survival was obtained by relating cfu 2, 4, 24, and 48 hours post infection to cfu 0.5 hours post infection. Viability of RAW264.7 cells was determined using the live/dead viability kit (life technologies, U.S.) according to manufacturer’s specifications. Briefly, the calcein live dye stains all cells, while the propidium iodide stains dead cells. Fluorescent images were acquired by exciting at 488 nm and thresholding of fluorescent images to determine cell outlines and counting of live and dead cells was performed using Fiji software^54^.

### Phagocytosis efficiency assays

The adhesion assays were performed in serum-free media to exclude potentially convolving effects from serum components. However, to confirm that serum did not influence the trend of the data, bacteria to macrophage ratio of 10:1 was analyzed also in serum-containing media (Supplementary Fig. S1a). The assay was conducted in the same manner as the gentamicin assay up to the incubation for 0.5 hours. Non-adherent bacteria were then removed by three washing steps with ice-cold phosphate buffered saline pH 7.4 (PBS) supplemented with 5 mM Ethylen-diamine-tetra-acetate (EDTA, Fluka 03677), after which cells were detached by vigorous pipetting with PBS-EDTA. Flow cytometric analysis was carried out on a BD LRS Fortessa and quantified using Flowjo V10 software (Treestar). The assay was repeated on 3 independent days with two biological replicates and two technical duplicates on each day.

### Live cell imaging

Automated live cell imaging of adhesion of *E.coli* to macrophages was performed in 3–15 minute intervals using a 40× objective and Nikon TE2000 (Egg, Switzerland) and Leica SP5 (Herbrugg, Switzerland) inverted microscopes in accordance with the experimental procedures of the gentamicin protection assay. Briefly, RAW264.7 macrophages were seeded on fibronectin coated cover glasses in custom made Polydimethylsiloxane (PDMS, Sylgard 184, Dow Corning, Midland, MI, USA) wells. Bacteria transformed with the P_*rpsM*_-GFP promoter fusion construct^53^ were added to the respective bacteria to macrophage ratio and incubated at 37 °C for 0.5 hours. Media was then replaced for 0.25 hours to serum free media supplemented with 100 μg/ml gentamicin (Sigma 48760-1G-F, Buchs, Switzerland) and after that to media supplemented additionally with 10% FBS and 10 μg/ml gentamicin for the cultivation during image acquisition.

### Lysosomal acidification assay

To assess lysosomal acidification, the commercially available LysoID kit (ENZ-51005–500) was used according to the manufacturer specifications. Briefly, RAW264.7 macrophages were seeded on fibronectin coated cover glasses in custom made Polydimethylsiloxane (PDMS, Sylgard 184, Dow Corning, Midland, MI, USA) wells. Bacteria transformed with the P_*rpsM*_-GFP promoter fusion construct^53^ were added to the respective bacteria to macrophage ratio and incubated at 37 °C for 0.5 hours. Media was then replaced for 0.25 hours with LysoID buffer containing the LysoID Red detection reagent and the Hoechst nuclear stain 33342, and washed afterwards with LysoID assay buffer prior to confocal imaging. 20 z-slices (0.34 μm steps) were used for a projection of maxium intensities. A region of interest containing stained compartments and bacteria was measured using the ImageJ histogram function and the mean of the histogram was plotted.

### Statistical analysis

Descriptive statistics of mean values, standard deviation (S.D.) and one-way ANOVA tests of single cell and population data were performed using Origin 9.0 software (OriginLab, Northhampton, MA, USA). Statistical significance was estimated using the post hoc Tukey and Bonferroni Tests at the specified thresholds and grouping of the samples based on how different their means were. The post hoc Tukey test was used to determine homogeneous subsets of samples, that is, groups of samples that are not significantly different from each other at a specified significance level alpha. Homogeneous subsets were labeled with upper case letters in the Figures. Samples that belong to different homogeneous subsets are marked with more than one letter. Pairs of samples that have no letter in common have significantly different means; pairs of samples that have one or more letter in common do not have significantly different means. Predictive statistics for fitting was performed using Origin 9.0 software and the built-in Michaelis Menten formula as well as custom-build functions for a one-step irreversible model (for details, see Supplementary Information, equations (2)–(12)). An overview on all actual p-Values in found in the Supplementary Information of this manuscript (Supplementary Tables T1–T8).

## Additional Information

**How to cite this article**: Avalos Vizcarra, I. *et al.* How type 1 fimbriae help *Escherichia coli* to evade extracellular antibiotics. *Sci. Rep.* **6**, 18109; doi: 10.1038/srep18109 (2016).

## Supplementary Material

Supplementary Movie M1

Supplementary Movie M2

Supplementary Information
