# Supplementary material for: How type 1 fimbriae help Escherichia coli to evade extracellular antibiotics
Source: Sci Rep. 2016 Jan 5;6:18109. doi: 10.1038/srep18109 (PMC4700443; doi:10.1038/srep18109)
Supplement: Supplementary Information [file srep18109-s3.pdf]

Supplementary information

How type 1 fimbriae help *Escherichia coli* to evade extracellular antibiotics

Ima Avalos Vizcarra[1], Vahid Hosseini[1], Philip Kollmannsberger[1], Stefanie Meier[1], Stefan S. Weber[2], Markus Arnoldini[3,4,#], Martin Ackermann[3,4], Viola Vogel[1*]

The use of serum-containing media did not change the fimbriae-mediated higher adhesion to macrophages

The experimental data on macrophage-bacteria adhesion were obtained under the assumption that adhesion of fimbriae-expressing, pathogenic *E.coli* occurs under serum-free conditions, such as in the urinary tract where uropathogenic *E.coli* are found. The adhesion assays in this study were thus performed in serum free media. To make sure that the serum free adhesion assays did not interfere with the infection efficiencies of macrophages by *E.coli*, we performed the experiments in the same cell culture media with 10% FBS at a bacteria to macrophage ratio of 10, yielding the same trends as in serum-free media, although differences in the total amounts of macrophages were observed (Supplementary Figure S1a). For Δfim, the difference in adhesion efficiencies when serum was present amounted roughly to a factor of three. This finding is consistent with the known role of LPS binding protein (LBP), which enhances adhesion of LPS-positive bacteria. Serum-free conditions mimic non-opsonic environment, e.g. in the urinary tract where no opsonization by complement or other serum components can occur.

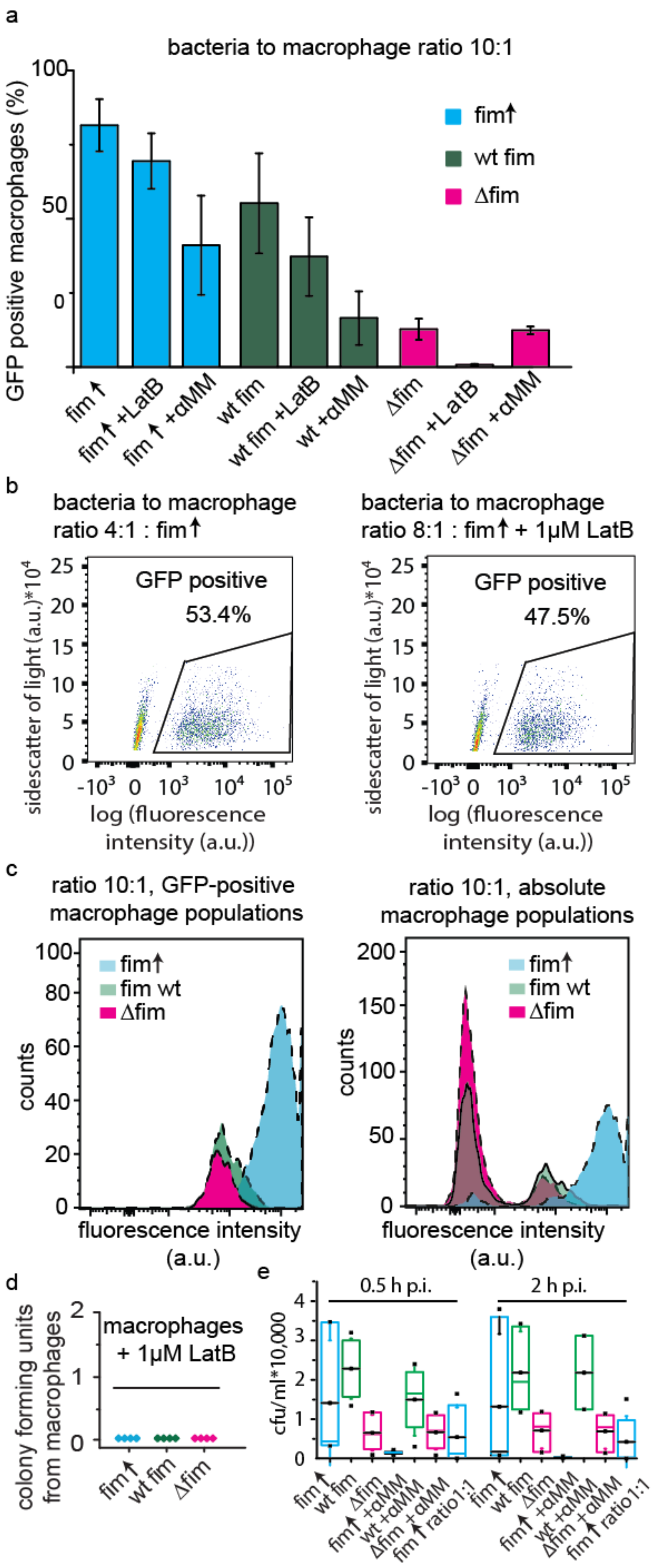
a
bacteria to macrophage ratio 10:1
GFP positive macrophages (%)
100
50
0
fim↑
wt fim
Δfim
fim↑
fim↑ +LatB
fim↑ +αMM
wt fim
wt fim +LatB
wt +αMM
Δfim
Δfim +LatB
Δfim +αMM
b
bacteria to macrophage ratio 4:1 : fim↑
bacteria to macrophage ratio 8:1 : fim↑ + 1μM LatB
GFP positive
53.4%
47.5%
sidescatter of light (a.u.)*10^4
log (fluorescence intensity (a.u.))
c
ratio 10:1, GFP-positive macrophage populations
ratio 10:1, absolute macrophage populations
fim↑
fim wt
Δfim
counts
fluorescence intensity (a.u.)
d
colony forming units from macrophages
macrophages + 1μM LatB
fim↑
wt fim
Δfim
e
0.5 h p.i.
2 h p.i.
cfu/ml*10,000
fim↑
wt fim
Δfim
fim↑ +αMM
wt +αMM
Δfim +αMM
fim↑ ratio1:1

Supplementary Figure S1: Type 1 fimbriae cause higher bacterial adhesion to macrophages also in the presence of serum and internalization inhibitors. (a) Adhesion efficiencies in serum-containing media follow the same trend as in serum-free media. Surface adherent macrophages were challenged with GFP-expressing bacteria at a bacteria-to-macrophage ratio of 10:1 for 0.5 hours in DMEM media supplemented with 10% FBS. Macrophages were then detached from the surface and the fraction of GFP-positive macrophages was quantified by flow cytometry as described in the main text of this manuscript and the material and methods section of the main manuscript. (b) Treatment with LatB did not abolish bacterial adhesion to macrophages. Original flow cytometry plots for two randomly chosen samples of macrophage incubation with fim and fim & 1 μM LatB, respectively. (c) Intensity histograms of GFP positive macrophages incubated with GFP-expressing fim↑, wt fim and Δfim *E.coli*, showed higher fluorescence intensity for macrophages incubated with fimbriated bacteria at the same ratio of 10:1. (d) Lysates of LatB-treated macrophages yielded no surviving *E.coli*, indicating that internalization was required for bacterial survival. cfu, colony forming units; fim↑, fimbriae overexpression strain; wt, fimbriae wild type strain; Δfim, fimbriae knockout strain; GFP, green fluorescent protein; p.i., post infection; αMM, alpha-methyl mannosepyrannoside; LatB, LatrunculinB; h, hours.

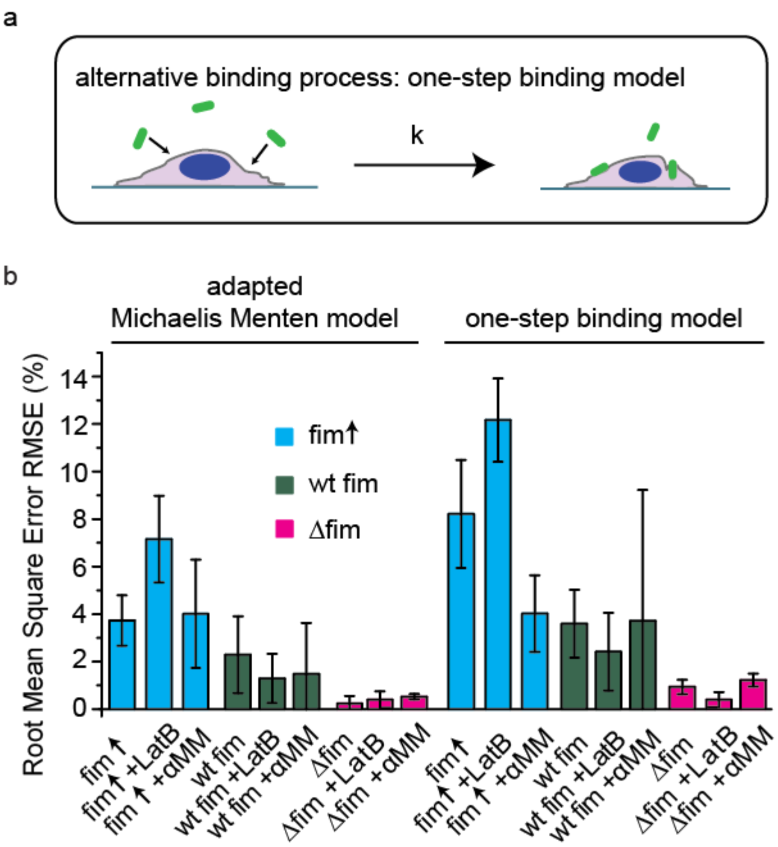

Supplementary Figure S2: An alternative one-step model fits the data less well than the adapted Michaelis Menten model. (a) Binding scheme of an irreversible binding model, following an exponential binding behavior (Eq. 2). (b) Root mean square error (RMSE) values of the two-step, reversible and one-step, irreversible model for bacterial adhesion. An RMSE value was obtained as an estimate of the fit performance for each adhesion curve and the respective fit from the adapted Michaelis Menten (Eq. 1) and the exponential adhesion model (Eq. 2). Error bars are S.D. fim↑, fimbriae overexpression strain; wt, fimbriae wild type strain; Δfim, fimbriae knockout strain; αMM, alpha-methyl mannosepyrannoside; LatB, LatrunculinB.

Modeling the adhesion kinetics of bacteria to macrophages

a) **Michaelis Menten model: Assuming a reversible, non-cooperative two-step internalization process**

From the observation of a saturation trend in the adhesion of bacteria to macrophages for the fimbriae overexpression strain (Fig. 2b), we tested if Michaelis Menten-like kinetics could describe the experimentally determined adhesion data. Cooperativity is an important factor in binding kinetics and has been shown e.g. in the infection of epithelial and HeLa cells by Salmonella[1]. Characteristic binding curves of cooperative processes show a sigmoidal dose response curve, a feature that we did not observe in our data. We thus tested a non-cooperative Michaelis-Menten model for its ability to fit the experimental dose response data. Accordingly, initial adhesion of bacteria to macrophages was assumed to be reversible, such that physical contact between bacteria and macrophage can result in transient, weak adhesion at a rate k_{on} with unbinding at a rate k_{off}. In a subsequent step, bacteria can stably adhere to the macrophage surface and be taken up in a slower, rate-limiting step at a rate k. The rate limiting and irreversible step results in the GFP-positive macrophage population which can be detected by flow cytometry.

$$MP + B \underset{k_{off}}{\overset{k_{on}}{\rightleftarrows}} (MP - B)_{inst} \xrightarrow{k} (MP - B)_{stable} \quad (2)$$

where MP = macrophage, B = bacterium,$[MP\text{-}B]_{inst}$ = macrophages with one or more instably bound bacteria and $[MP\text{-}B]_{stable}$ = macrophages with one or more stably bound bacteria. MP in this complex can bind additional bacteria.

Assuming that weak adhesion and unbinding are in instantaneous equilibrium, the concentration of free bacteria does not change over time. Consequently,

$$k_{on} * [MP] * [B] = k_{off} * [MP - B]_{inst} \quad (3)$$

Using the definition

$$[\mathrm{MP}] + [MP - B]_{inst} = [MP]_{total} \quad (4)$$

for all undetected MP, this can be rewritten as

$$[MP - B]_{inst} = \frac{[MP]_{total} * [B]}{(K_s + [B])} \quad (5)$$

where

$$K_s = \frac{k_{off}}{k_{on}} \quad (6)$$

From this, the velocity M of conversion of macrophages with bacteria bound $[MP\text{-}B]_{inst}$ to detectable infected macrophages $[MP\text{-}B]_{stable}$ can be derived as

$$M = \frac{d[MP - B]_{stable}}{dt} = k * [MP - B]_{inst} = \frac{k * [MP]_{total} * [B]}{(K_s + [B])}$$
$$= M_{max} \frac{[B]}{(K_s + [B])} \quad (7)$$

where

$$M_{max} = k * [MP - B]_{total} \quad (8)$$

is the maximum reaction velocity under saturation when all macrophages have weakly bound bacteria. We measured $[MP\text{-}B]_{stable}/[MP]_{total}$ at a fixed time t_0 for different [B], i.e. bacteria to macrophage ratios, which gives the velocities M since $[MP\text{-}B]_{tota\ l} \sim M^* t_0$. Therefore, we can fit the resulting concentrations as a function of [B] using the equation above, even though we did not measure M directly.

Note that the K_s from the fit is not a rate per se, but a dimensionless ratio of two rates which is inversely proportional to the binding rate in the exponential model, since $K_s \sim 1/k_{on}$. The maximum amount of macrophages that can be bound was set to an upper limit of 100%.

b) The one-step adhesion model: Assuming an irreversible, non-cooperative internalization process:

In an alternative approach on modeling the rate of bacterial adhesion, we tested the assumption that the adhesion of bacteria is irreversible, i.e. contact between receptors and bacterial ligands immediately leads to formation of a stable complex that results in phagocytosis such that dissociation of the receptor-ligand complex is not possible.

$$R(x) = M_{max} * \left(1 - e^{(-k*t*x)}\right) \quad (9)$$

with the rate of internalization R(x), the bacteria to macrophage ratio x, the adhesion on-rate k and the time t.

Furthermore, a non-cooperative adhesion process was assumed, since the binding curve obtained from the raw data did not show a sigmoidal behavior as had been observed for example for Salmonella mediated infection of epithelial cells[1]. In this case one would also expect to observe a sigmoidal shape of the binding curve. In analogy to a reaction scheme:

$$MP + B \xrightarrow{k} [MP - B]_{stable} \tag{10}$$

The change of the fraction of infected macrophages r depends on the bacteria to macrophage ratio x, and the bacterial adhesion rate k, and the fraction of not yet infected macrophages (1-r):

$$\frac{dr}{dt} = k * x * (1 - r) \tag{11}$$

This ordinary differential equation has the solution

$$r(t) = 1 - e^{(-k*x*t)} \tag{12}$$

Since x, but not t was varied in the experimental design, a resulting fit using this exponential expression will give the parameter k. The root mean square error (RMSE) values of both models showed that the deviation between fit and experimental data was lower for the Michaelis Menten model (Supplementary Fig. S2b).

The fit parameters are shown in the Figure 3d and 3e of the main text. The absolute numbers are not related to experimental parameters since the flow cytometric assay did not quantify the number of bacteria per macrophage, or the number of adhesion attempts per time as only fixed timepoints were available. Under the assumptions we used, the purpose of the model is to estimate differences in adhesion rates for the different proposed steps, information that would otherwise not be experimentally accessible.

Statistical analysis

For an overview on all statistical tests that we applied in this study, the results from the one-way ANOVA and one-tailed t-test are found in the Supplementary tables T1-T8.

Supplementary Table T1: Parameters from the post-hoc Bonferroni test of the one-way ANOVA (used for Fig. 1c)

Condition 1-Condition 2	MeanDiff	SEM	t Value	Probability	Alpha	Significance
wt - fim↑	-1,811,666,667	325,103,545	-557,258	1.60E+01	0,01	1
Δfim - fim↑	-25325	325,103,545	-778,983	3.58E-01	0,01	1
Δfim - wt	-720,833,333	325,103,545	-221,724	0,12742	0,01	0

Supplementary Table T2: Parameters from the post-hoc Bonferroni test of the one-way ANOVA (used for Fig. 1d)

Condition 1 – Condition 2	MeanDiff	SEM	t Value	Probability	Alpha	Significance
**4 hours post infection**						
wt - fim↑	-0,56305	0,11561	-487,006	2.45E-04	0,01	1
Δfim - fim↑	-0,45145	0,11561	-390,485	0,00245	0,01	1
Δfim - wt	0,11159	0,11561	0,96521	1	0,01	0
**24 h post infection**						
wt - fim↑	-0,60491	0,2196	-275,459	0,03117	0,01	0
Δfim - fim↑	-0,77047	0,2196	-350,848	0,00479	0,01	1
Δfim - wt	-0,16555	0,2196	-0,75389	1	0,01	0

Supplementary Table T3: Parameters from the post-hoc Bonferroni test of the one-way ANOVA (used for Fig. 1e)

Condition 1 – Condition 2	MeanDiff	SEM	t Value	Probability	Alpha	Significance
pos. Ctrl 100uM - Chloroquine neg Ctrl	44.30367	6.11213	7.24848	1.34E-09	0.01	1
Δfim - neg Ctrl	-0.83991	6.40335	-0.13117	1	0.01	0
Δfim - pos. Ctrl 100uM Chloroquine	-45.1436	4.86729	-9.27488	8.40E-14	0.01	1
Fim↑ - neg Ctrl	-8.554	6.5092	-1.31414	1	0.01	0
Fim↑ - pos. Ctrl 100uM Chloroquine	-52.8577	5.00573	-10.5594	1.74E-16	0.01	1
Fim↑ - Δfim	-7.71409	5.35743	-1.43989	1	0.01	0
Fim↑3MA - neg Ctrl	-10.0882	7.10388	-1.4201	1	0.01	0
Fim↑3MA - pos. Ctrl 100uM Chloroquine	-54.3919	5.75784	-9.44658	3.67E-14	0.01	1
Fim↑3MA - Δfim	-9.24832	6.06609	-1.52459	1	0.01	0
Fim↑3MA - Fim↑	-1.53423	6.17772	-0.24835	1	0.01	0

Supplementary Table T5: Parameters from the post-hoc Bonferroni test of the one-way ANOVA (used for Fig. 2h)

Condition 1 – Condition 2	MeanDiff	SEM	t Value	Prob	Alpha	Sig
wt - fim↑	-0.48561	0.20254	-2.39765	1	0.05	0
Δfim - fim↑	-0.72773	0.20254	-3.59308	0.05185	0.05	0
Δfim - wt	-0.24212	0.20254	-1.19544	1	0.05	0
fim↑ LatB - fim↑	-1.61957	0.20254	-7.99646	2.85E-07	0.05	1
fim↑ LatB - wt	-1.13396	0.20254	-5.59881	1.93E-04	0.05	1
fim↑ LatB - Δfim	-0.89184	0.20254	-4.40338	0.00561	0.05	1
wt LatB - fim↑	-1.61957	0.20254	-7.99646	2.85E-07	0.05	1
wt LatB - wt	-1.13396	0.20254	-5.59881	1.93E-04	0.05	1
wt LatB - Δfim	-0.89184	0.20254	-4.40338	0.00561	0.05	1
wt LatB - fim↑ LatB	0	0.20254	0	1	0.05	0
Δfim LatB - fim↑	-1.61957	0.20254	-7.99646	2.85E-07	0.05	1
Δfim LatB - wt	-1.13396	0.20254	-5.59881	1.93E-04	0.05	1
Δfim LatB - Δfim	-0.89184	0.20254	-4.40338	0.00561	0.05	1
Δfim LatB - fim↑ LatB	0	0.20254	0	1	0.05	0
Δfim LatB - wt LatB	0	0.20254	0	1	0.05	0
fim↑ amm - fim↑	-0.74457	0.20254	-3.67623	0.04148	0.05	1
fim↑ amm - wt	-0.25896	0.20254	-1.27858	1	0.05	0
fim↑ amm - Δfim	-0.01684	0.20254	-0.08314	1	0.05	0
fim↑ amm - fim↑ LatB	0.875	0.20254	4.32023	0.00708	0.05	1
fim↑ amm - wt LatB	0.875	0.20254	4.32023	0.00708	0.05	1
fim↑ amm - Δfim LatB	0.875	0.20254	4.32023	0.00708	0.05	1
wt amm - fim↑	-0.53598	0.20254	-2.64633	0.57751	0.05	0
wt amm - wt	-0.05037	0.20254	-0.24868	1	0.05	0
wt amm - Δfim	0.19175	0.20254	0.94675	1	0.05	0
wt amm - fim↑ LatB	1.08359	0.20254	5.35013	3.90E-04	0.05	1
wt amm - wt LatB	1.08359	0.20254	5.35013	3.90E-04	0.05	1
wt amm - Δfim LatB	1.08359	0.20254	5.35013	3.90E-04	0.05	1
wt amm - fim↑ amm	0.20859	0.20254	1.0299	1	0.05	0
Δfim amm - fim↑	-0.76244	0.20254	-3.76449	0.03268	0.05	1
Δfim amm - wt	-0.27683	0.20254	-1.36684	1	0.05	0
Δfim amm - Δfim	-0.03472	0.20254	-0.17141	1	0.05	0
Δfim amm - fim↑ LatB	0.85712	0.20254	4.23197	0.00905	0.05	1
Δfim amm - wt LatB	0.85712	0.20254	4.23197	0.00905	0.05	1
Δfim amm - Δfim LatB	0.85712	0.20254	4.23197	0.00905	0.05	1
Δfim amm - fim↑ amm	-0.01788	0.20254	-0.08826	1	0.05	0
Δfim amm - wt amm	-0.22647	0.20254	-1.11816	1	0.05	0
fim↑ RATIO 1 - fim↑	-0.46542	0.20254	-2.29795	1	0.05	0
fim↑ RATIO 1 - wt	0.02019	0.20254	0.0997	1	0.05	0
fim↑ RATIO 1 - Δfim	0.26231	0.20254	1.29513	1	0.05	0
fim↑ RATIO 1- fim↑ LatB	1.15415	0.20254	5.69851	1.46E-04	0.05	1
fim↑ RATIO 1 - wt LatB	1.15415	0.20254	5.69851	1.46E-04	0.05	1

Supplementary Table T5 Δ(continued): Parameters from the post-hoc Bonferroni test of the one-way ANOVA (used for Fig. 2h)

fim↑ RATIO 1 - Δfim LatB	1.15415	0.20254	5.69851	1.46E-04	0.05	1
fim↑ RATIO 1 - fim↑ amm	0.27915	0.20254	1.37828	1	0.05	0
fim↑ RATIO 1 - wt amm	0.07056	0.20254	0.34838	1	0.05	0
fim↑ RATIO 1 - Δfim amm	0.29703	0.20254	1.46654	1	0.05	0

Supplementary Table T6: Parameters from the post-hoc Bonferroni test of the one-way ANOVA (used for Fig. 4b)

Condition 1 – Condition 2	MeanDiff	SEM	t Value	Probability	Alpha	Sig
fim↑ 0.5 h control 0.5 h	-0.95241	1.66504	-0.57201	1	0.05	0
wt 0.5 h control 0.5 h	-0.03937	1.66504	-0.02365	1	0.05	0
wt 0.5 h fim↑ 0.5 h	0.91304	1.66504	0.54836	1	0.05	0
Δfim 0.5 h control 0.5 h	3.76112	1.66504	2.25887	1.00E+00	0.05	0
Δfim 0.5 h fim↑ 0.5 h	4.71353	1.66504	2.83088	6.10E-01	0.05	0
Δfim 0.5 h wt 0.5 h	3.80049	1.66504	2.28252	1	0.05	0
control 24 h control 0.5 h	5.2872	1.66504	3.17542	2.69E-01	0.05	0
control 24 h fim↑ 0.5 h	6.23961	1.66504	3.74743	6.57E-02	0.05	0
control 24 h wt 0.5 h	5.32657	1.66504	3.19907	0.25406	0.05	0
control 24 h Δfim 0.5 h	1.52609	1.66504	0.91655	1	0.05	0
fim↑ 24 h control 0.5 h	7.30008	1.66504	4.38433	1.31E-02	0.05	1
fim↑ 24 h fim↑ 0.5 h	8.25249	1.66504	4.95633	3.06E-03	0.05	1
fim↑ 24 h wt 0.5 h	7.33945	1.66504	4.40797	0.01235	0.05	1
fim↑ 24 h Δfim 0.5 h	3.53896	1.66504	2.12545	1	0.05	0
fim↑ 24 h control 24 h	2.01288	1.66504	1.20891	1	0.05	0
wt 24 h control 0.5 h	6.60029	1.66504	3.96404	0.03808	0.05	1
wt 24 h fim↑ 0.5 h	7.55271	1.66504	4.53605	0.00892	0.05	1
wt 24 h wt 0.5 h	6.63967	1.66504	3.98769	0.03587	0.05	1
wt 24 h Δfim 0.5 h	2.83918	1.66504	1.70517	1	0.05	0
wt 24 h control 24 h	1.31309	1.66504	0.78862	1	0.05	0
wt 24 h fim↑ 24 h	-0.69978	1.66504	-0.42028	1	0.05	0
Δfim 24 h control 0.5 h	6.76247	1.66504	4.06145	0.02977	0.05	1
Δfim 24 h fim↑ 0.5 h	7.71489	1.66504	4.63346	0.00696	0.05	1
Δfim 24 h wt 0.5 h	6.80185	1.66504	4.0851	0.02804	0.05	1
Δfim 24 h Δfim 0.5 h	3.00136	1.66504	1.80257	1.00E+00	0.05	0
Δfim 24 h control 24 h	1.47527	1.66504	0.88603	1.00E+00	0.05	0
Δfim 24 h fim↑ 24 h	-0.5376	1.66504	-0.32288	1.00E+00	0.05	0
Δfim 24 h wt 24 h	0.16218	1.66504	0.0974	1	0.05	0
control 48 h control 0.5 h	4.51027	1.66504	2.70881	0.80893	0.05	0
control 48 h fim↑ 0.5 h	5.46269	1.66504	3.28082	0.20831	0.05	0

Supplementary Table T6(continued): Parameters from the post-hoc Bonferroni test of the one-way ANOVA (used for Fig. 4b)

control 48 h wt 0.5 h	4.54965	1.66504	2.73246	0.76612	0.05	0
control 48 h Δfim 0.5 h	0.74916	1.66504	0.44993	1	0.05	0
control 48 h control 24 h	-0.77693	1.66504	-0.46661	1	0.05	0
control 48 h fim↑ 24 h	-2.7898	1.66504	-1.67552	1	0.05	0
control 48 h wt 24 h	-2.09002	1.66504	-1.25524	1	0.05	0
control 48 h Δfim 24 h	-2.2522	1.66504	-1.35264	1	0.05	0
fim↑ 48 h control 0.5 h	0.47354	1.66504	0.2844	1	0.05	0
fim↑ 48 h fim↑ 0.5 h	1.42595	1.66504	0.85641	1	0.05	0
fim↑ 48 h wt 0.5 h	0.51291	1.66504	0.30805	1	0.05	0
fim↑ 48 h Δfim 0.5 h	-3.28758	1.66504	-1.97447	1.00E+00	0.05	0
fim↑ 48 h control 24 h	-4.81366	1.66504	-2.89102	5.30E-01	0.05	0
fim↑ 48 h fim↑ 24 h	-6.82654	1.66504	-4.09992	2.70E-02	0.05	1
fim↑ 48 h wt 24 h	-6.12675	1.66504	-3.67964	0.0778	0.05	0
fim↑ 48 h Δfim 24 h	-6.28893	1.66504	-3.77705	0.06097	0.05	0
fim↑ 48 h control 48 h	-4.03673	1.66504	-2.42441	1	0.05	0
wt 48 h control 0.5 h	1.88104	1.66504	1.12973	1	0.05	0
wt 48 h fim↑ 0.5 h	2.83346	1.66504	1.70174	1	0.05	0
wt 48 h wt 0.5 h	1.92042	1.66504	1.15338	1	0.05	0
wt 48 h Δfim 0.5 h	-1.88007	1.66504	-1.12915	1	0.05	0
wt 48 h control 24 h	-3.40616	1.66504	-2.04569	1	0.05	0
wt 48 h fim↑ 24 h	-5.41903	1.66504	-3.2546	0.22203	0.05	0
wt 48 h wt 24 h	-4.71925	1.66504	-2.83432	0.60502	0.05	0
wt 48 h Δfim 24 h	-4.88143	1.66504	-2.93172	0.48144	0.05	0
wt 48 h control 48 h	-2.62923	1.66504	-1.57908	1	0.05	0
wt 48 h fim↑ 48 h	1.4075	1.66504	0.84533	1	0.05	0
Δfim 48 h control 0.5 h	5.19924	1.66504	3.12259	0.30554	0.05	0
Δfim 48 h fim↑ 0.5 h	6.15165	1.66504	3.6946	0.07495	0.05	0
Δfim 48 h wt 0.5 h	5.23861	1.66504	3.14624	0.28863	0.05	0
Δfim 48 h Δfim 0.5 h	1.43812	1.66504	0.86372	1	0.05	0
Δfim 48 h control 24 h	-0.08796	1.66504	-0.05283	1	0.05	0
Δfim 48 h fim↑ 24 h	-2.10084	1.66504	-1.26174	1	0.05	0
Δfim 48 h wt 24 h	-1.40106	1.66504	-0.84145	1	0.05	0
Δfim 48 h Δfim 24 h	-1.56324	1.66504	-0.93886	1	0.05	0
Δfim 48 h control 48 h	0.68896	1.66504	0.41378	1	0.05	0
Δfim 48 h fim↑ 48 h	4.7257	1.66504	2.83819	0.59958	0.05	0
Δfim 48 h wt 48 h	3.31819	1.66504	1.99286	1	0.05	0

Supplementary Table T7: Parameters from the post-hoc Bonferroni test of the one-way ANOVA (used for Fig. 4d)

Condition 1 - Condition 2	MeanDiff	SEM	t Value	Probability	Alpha	Significance
fim↑ - ctrl	127,408,476	5,892,596	2.16E+06	7.21E-86	1.00E-03	1
wt - ctrl	115,960,173	5,511,115	2,104,115	1.31E-81	1.00E-03	1
wt - fim↑	-11,448,303	6,418,714	-178,358	0,74669	1.00E-03	0
Δfim - ctrl	128,436,127	5,530,078	2,322,501	5.84E-98	1.00E-03	1
Δfim - fim↑	1,027,652	6,435,003	0,1597	1	1.00E-03	0
Δfim - wt	12,475,954	6,087,608	20,494	0,40575	1.00E-03	0
sup - ctrl	59,859,854	7,061,317	847,715	4.90E-11	1.00E-03	1
sup - fim↑	-67,548,621	779,036	-86,708	9.77E-12	1.00E-03	1
sup - wt	-56,100,318	7,505,958	-74,741	1.23E-07	1.00E-03	1
sup - Δfim	-68,576,273	7,519,892	-911,932	2.06E-13	1.00E-03	1

Supplementary Table T8: Parameters from the one-sample t-test (used for Fig. 4e)

Condition	t Statistic	DF	Prob>|t|
cells only	696,205	8	1.17E-04
Cells and bacteria	878,105	8	2.2E-05

Supplementary movie captions

Supplementary Movie M1: 3D reconstruction from confocal images of surface adherent macrophages incubated with fim↑ at a bacteria-to-macrophage ratio of 10. Bacteria were incubated with macrophages for 0.5 hours before fixing, staining and image acquisition.

Supplementary Movie M2: 3D reconstruction from confocal images of surface adherent macrophages incubated with Δfim at a bacteria-to-macrophage ratio of 10. Bacteria were incubated with macrophages for 0.5 hours before fixing, staining and image acquisition.

Supplementary references

1 Misselwitz, B. *et al.* Near Surface Swimming of Salmonella Typhimurium Explains Target-Site Selection and Cooperative Invasion. *PLoS Pathog.* **8**, doi:10.1371/journal.ppat.1002810 (2012).
